# Supplementary material for: Blood Clot Phenotyping by Rheometry: Platelets and Fibrinogen Chemistry Affect Stress-Softening and -Stiffening at Large Oscillation Amplitude
Source: Molecules. 2020 Aug 26;25(17):3890. doi: 10.3390/molecules25173890 (PMC7503632; doi:10.3390/molecules25173890)
Supplement: Supplementary file 1 [file molecules-25-03890-s001.zip › Supportive Figure 1.docx]

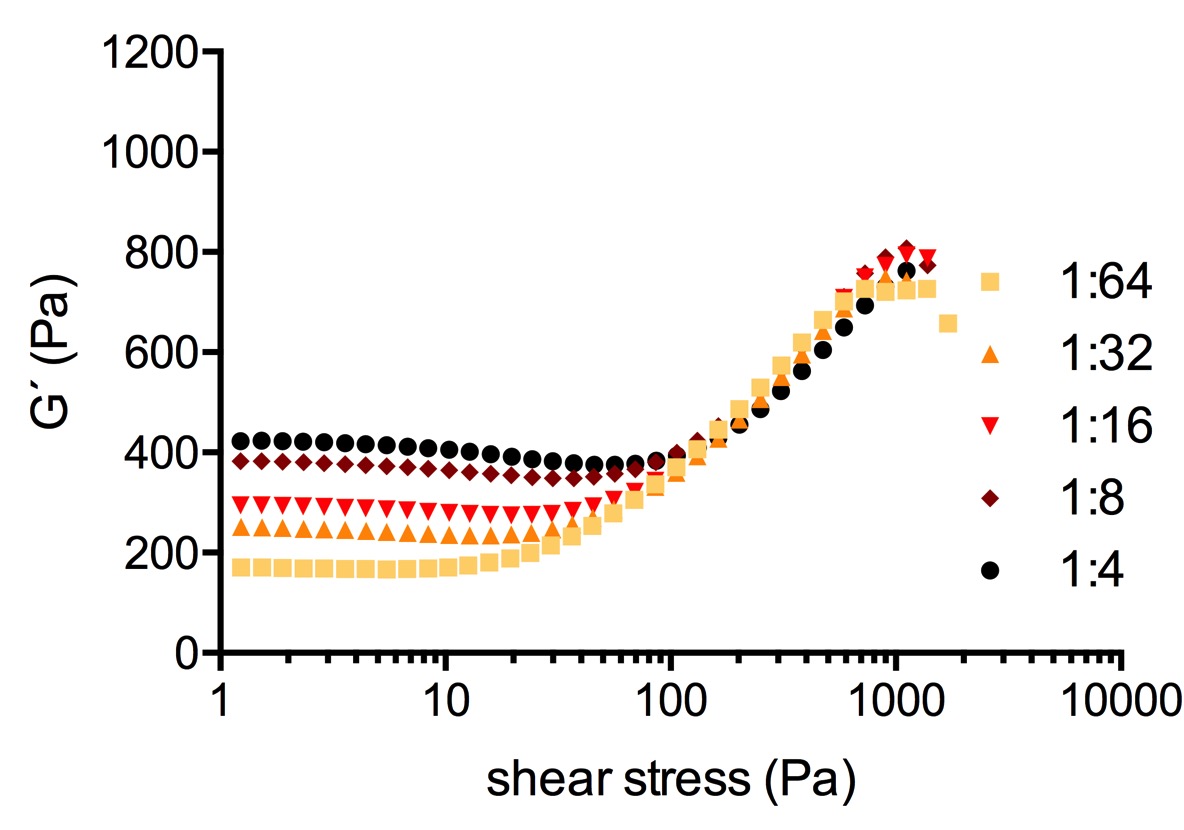

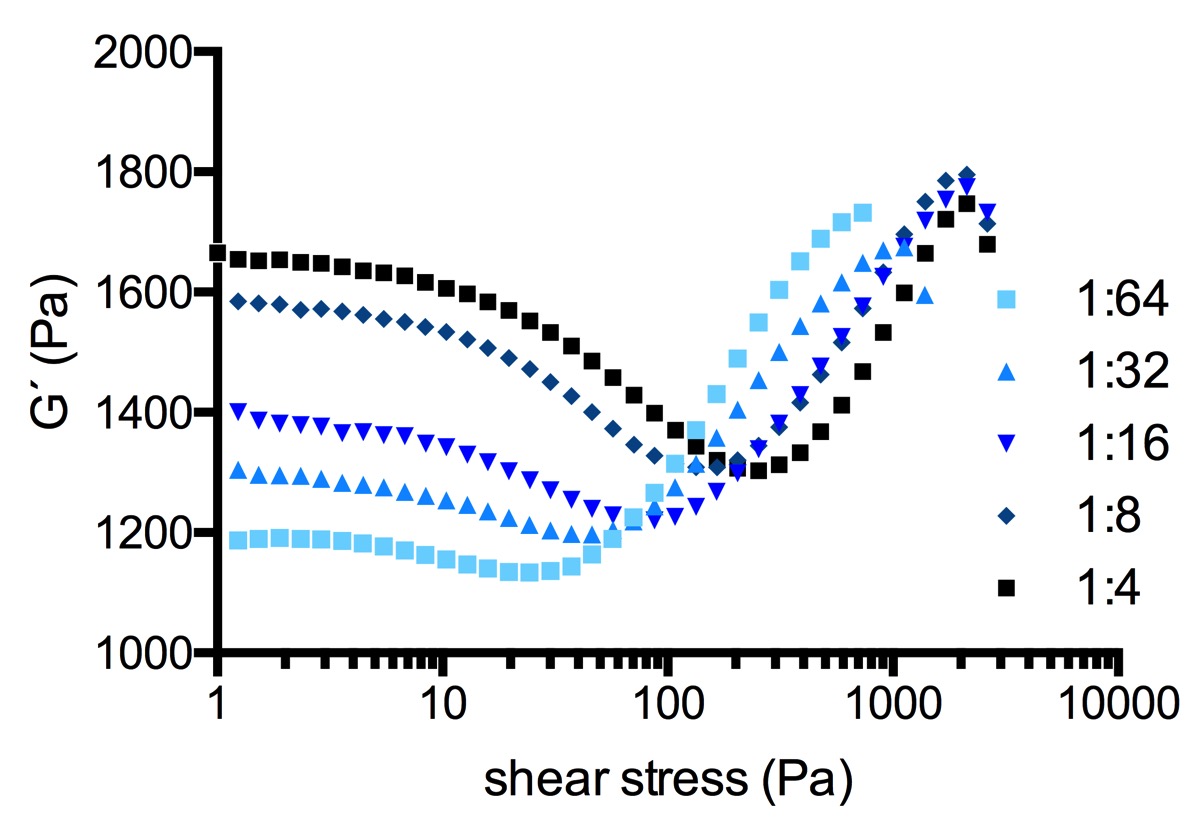

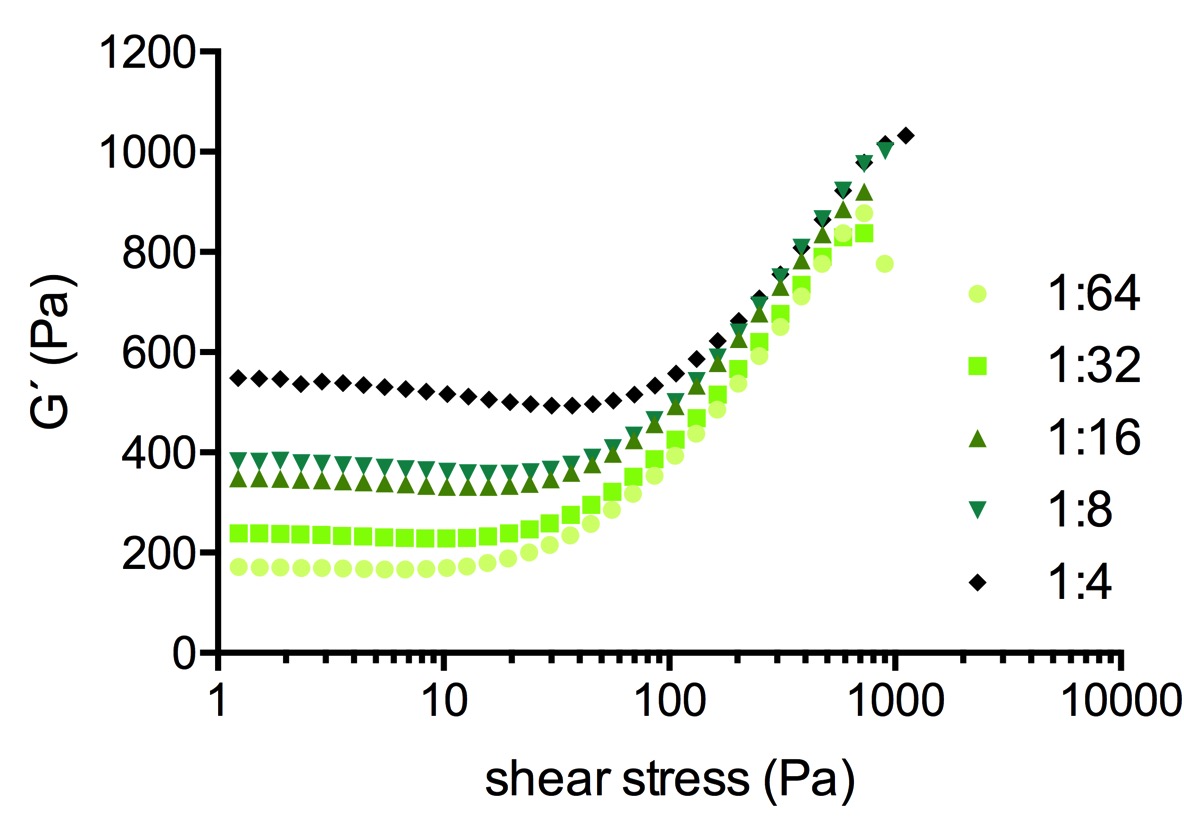

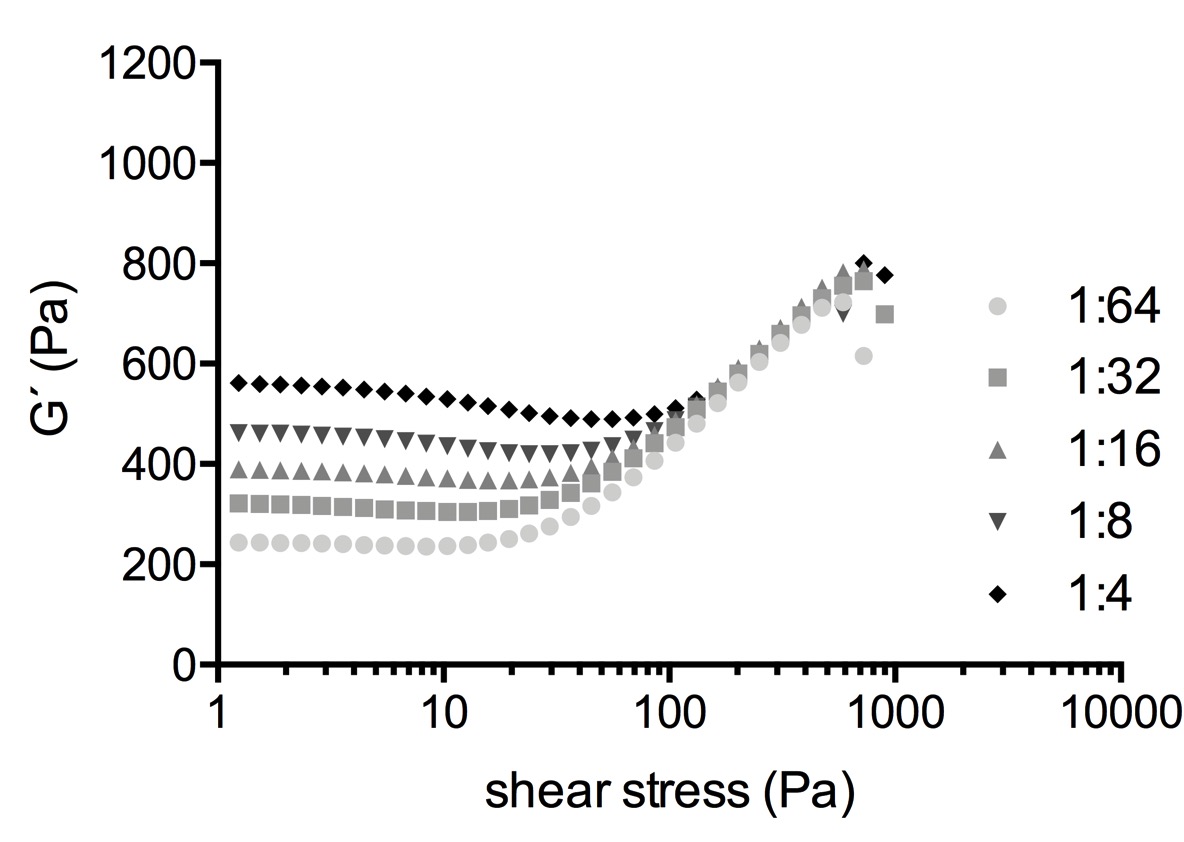


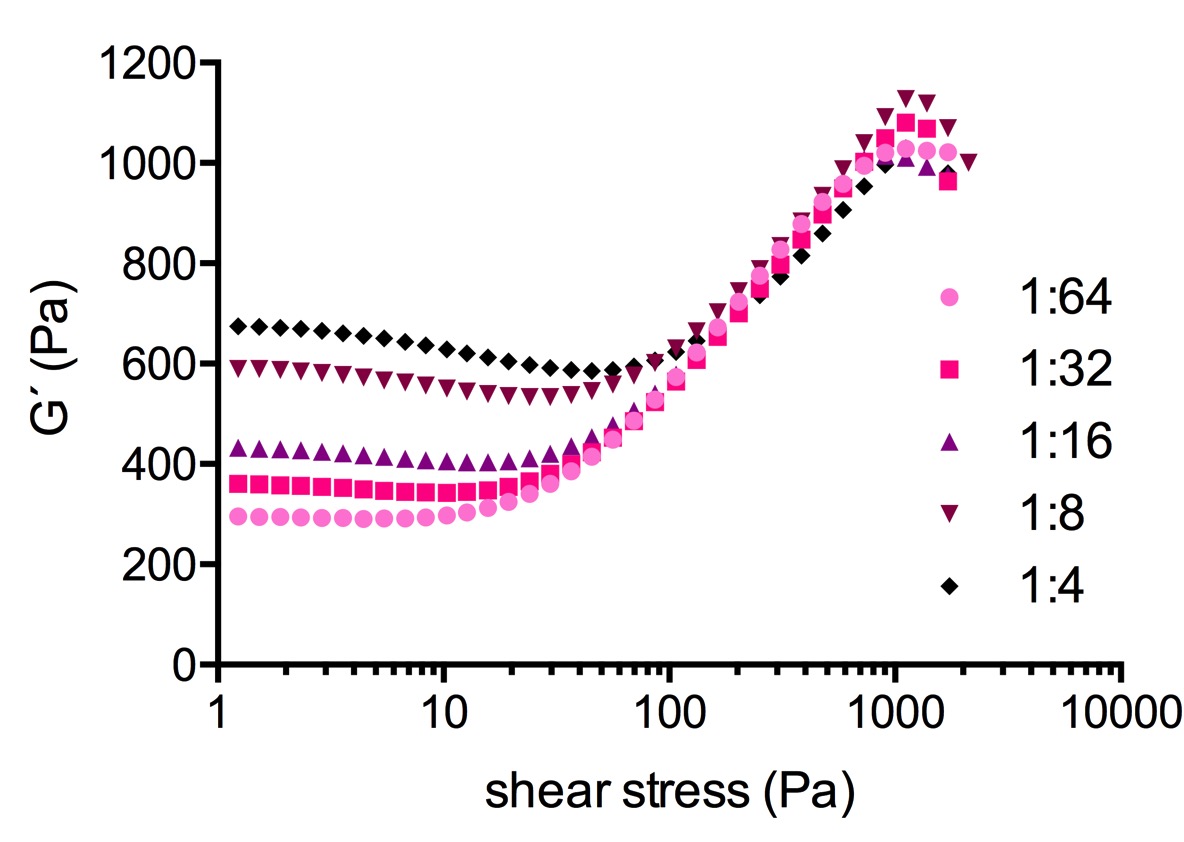


**Supportive Figure 1:** development of G´ during the LAOStress test (a: human, b: cow, c: swine, d: rat, f: horse). “1:64” marks a 1:64 dilution of platelet-rich plasma in platelet depleted plasma. Clot stiffness increases addition of platelets, but G ́ curves start to overlap at stresses >200 Pa in all tested species, except cow.
