# Supplementary material for: Blood Clot Phenotyping by Rheometry: Platelets and Fibrinogen Chemistry Affect Stress-Softening and -Stiffening at Large Oscillation Amplitude
Source: Molecules. 2020 Aug 26;25(17):3890. doi: 10.3390/molecules25173890 (PMC7503632; doi:10.3390/molecules25173890)
Supplement: Supplementary file 1 [file molecules-25-03890-s001.zip › Supportive Figure 3.docx]

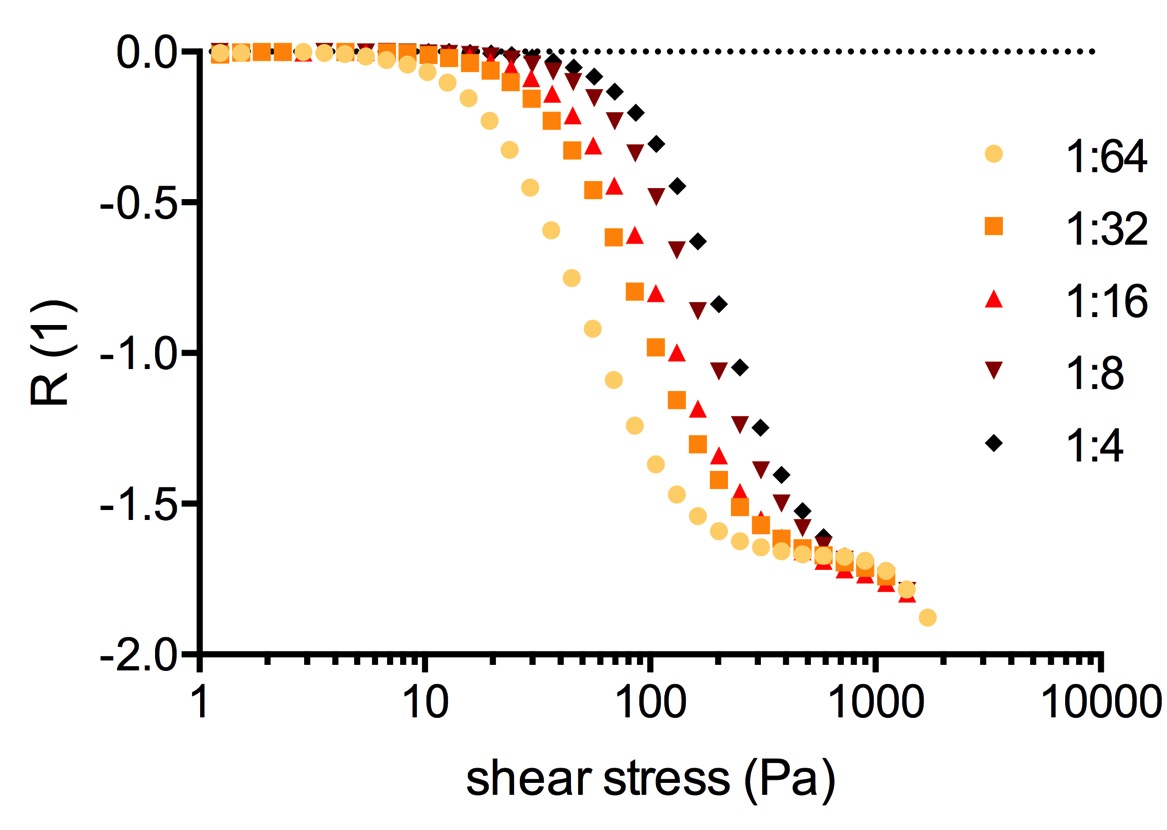

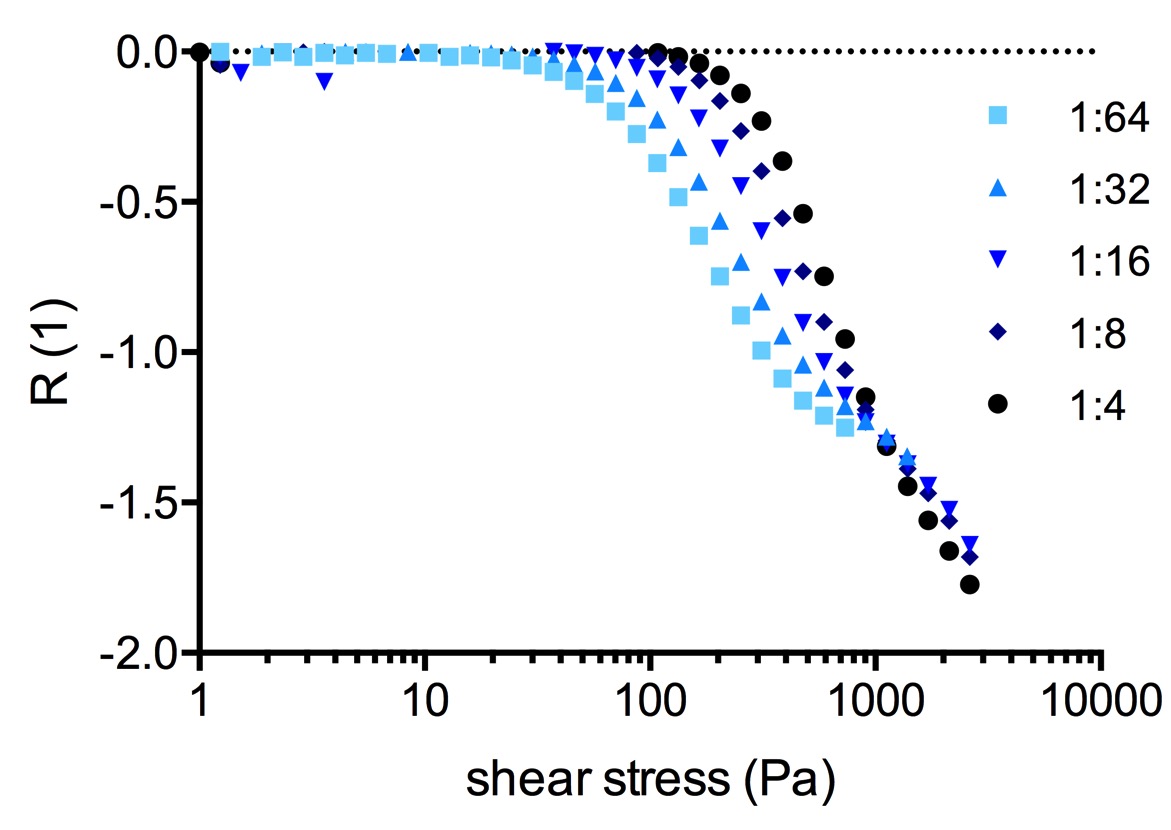

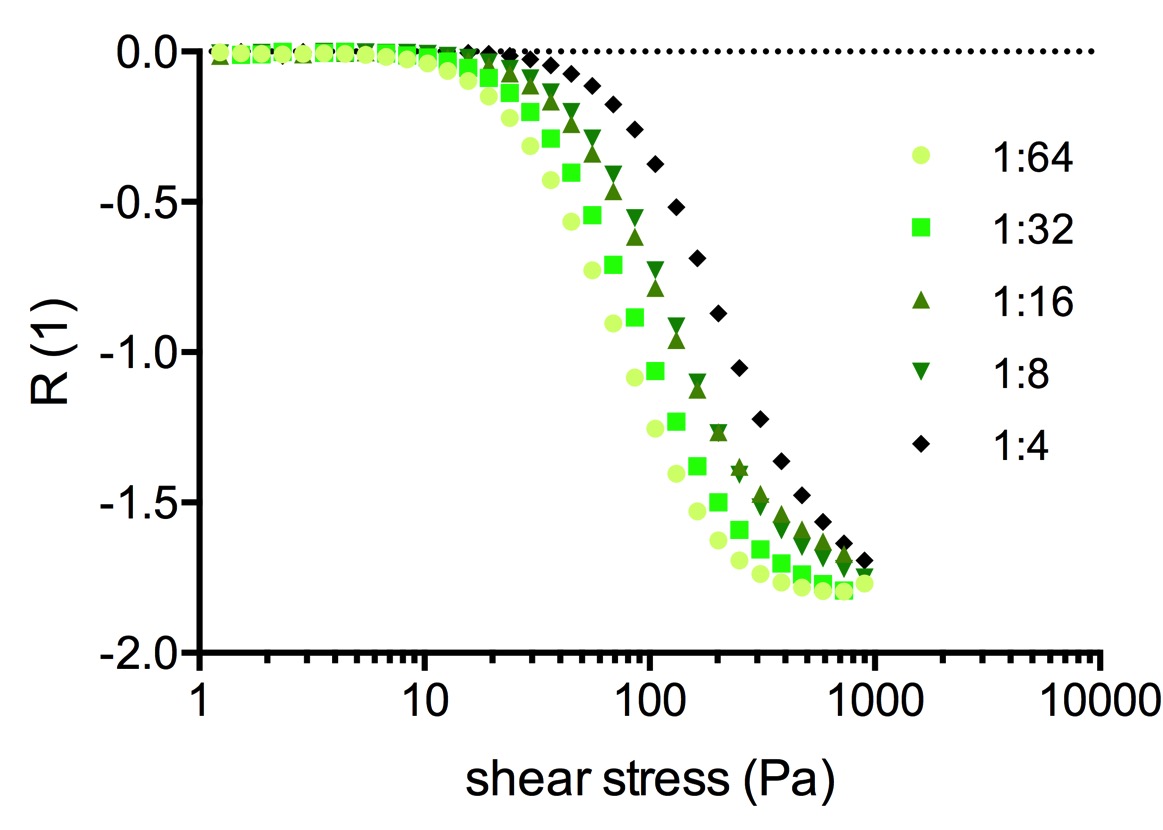

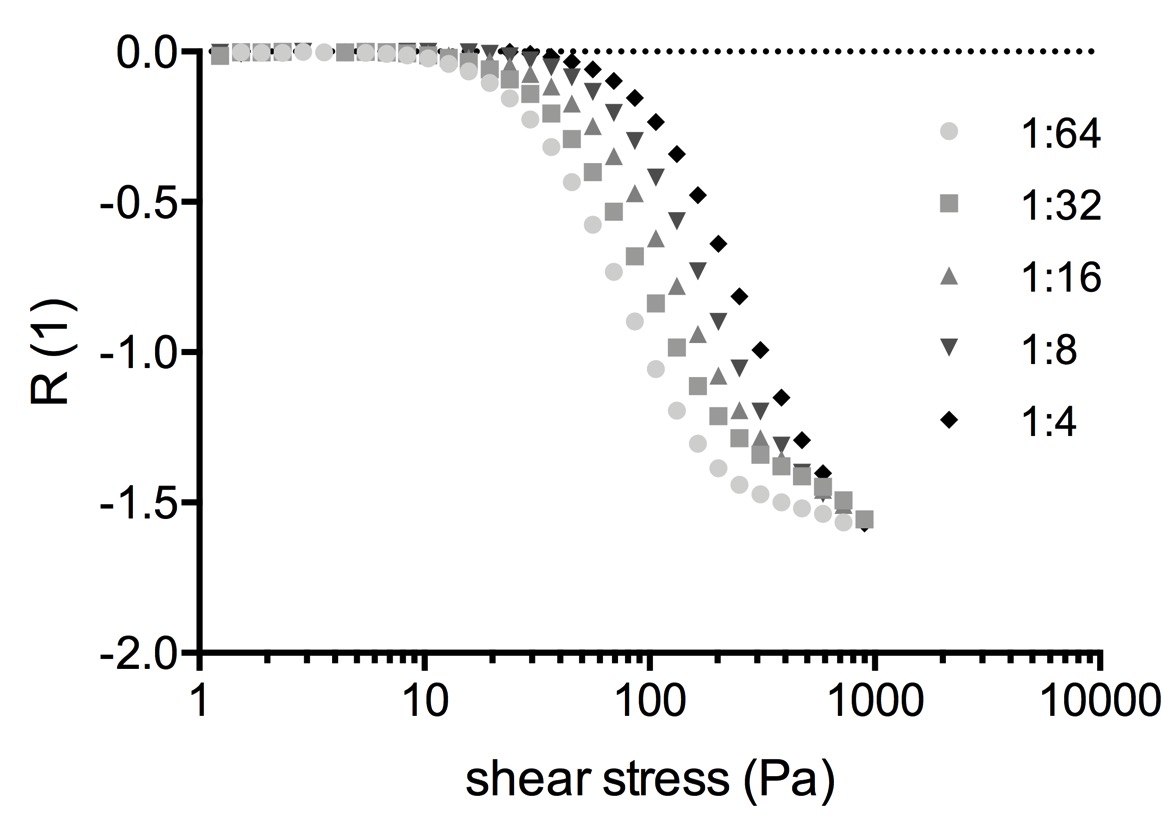


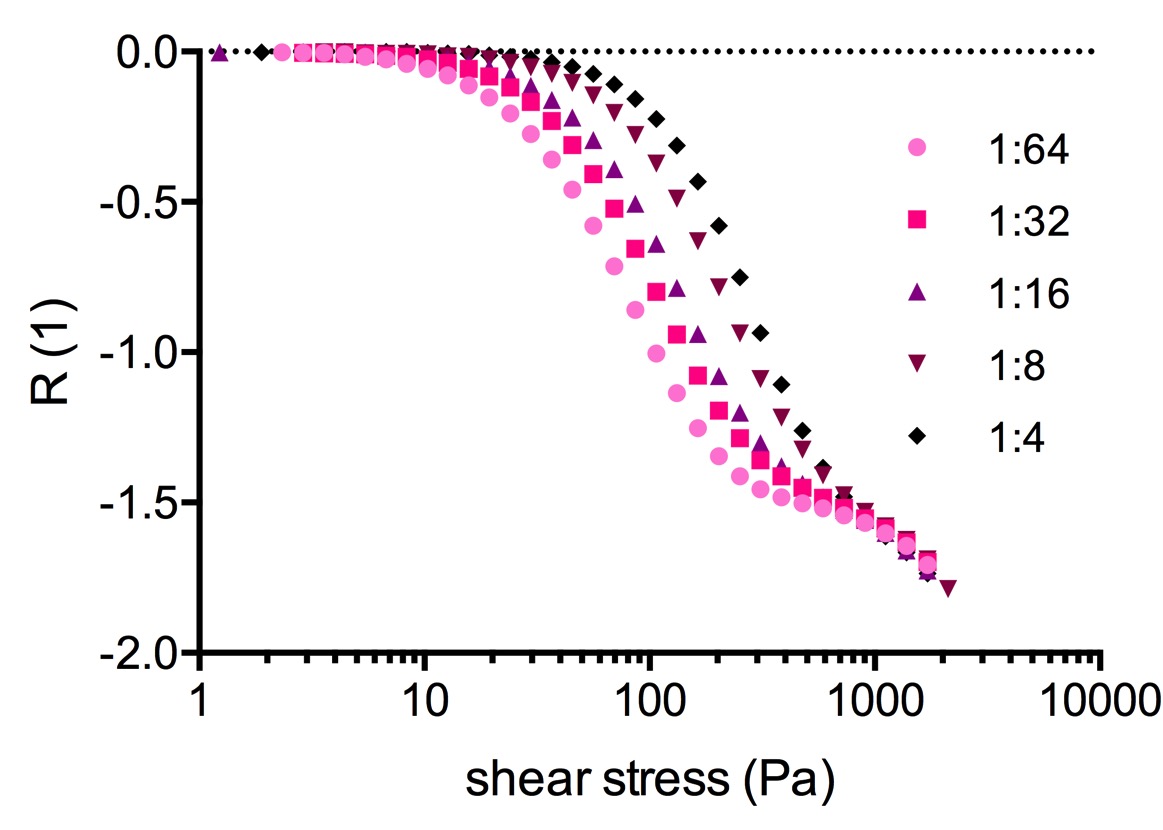


**Supportive Figure 3:** When the stress-softening ratio (R) becomes negative it indicates the onset of intra-cycle stiffening. R shifts to higher shear stress values in each species when platelets are added.
