# Supplementary material for: Blood Clot Phenotyping by Rheometry: Platelets and Fibrinogen Chemistry Affect Stress-Softening and -Stiffening at Large Oscillation Amplitude
Source: Molecules. 2020 Aug 26;25(17):3890. doi: 10.3390/molecules25173890 (PMC7503632; doi:10.3390/molecules25173890)
Supplement: Supplementary file 1 [file molecules-25-03890-s001.zip › Supportive Figure 2.docx]

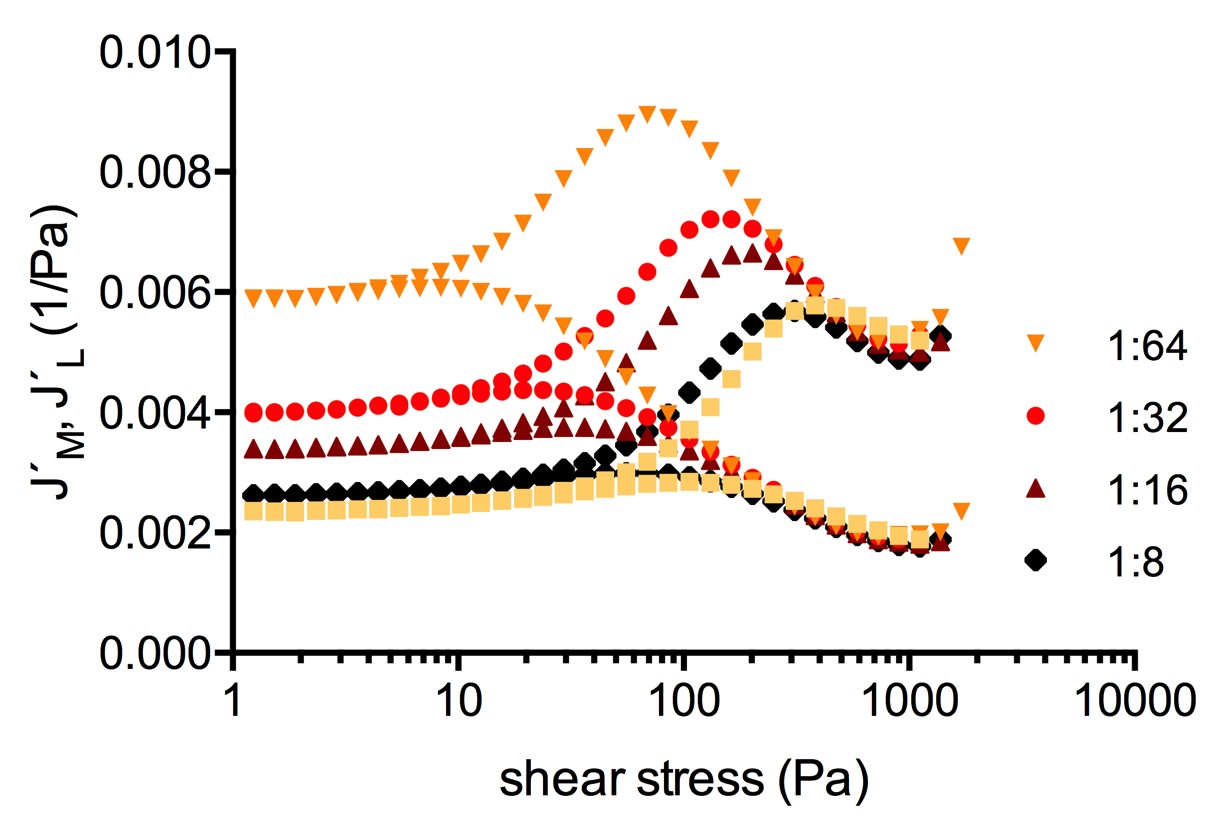

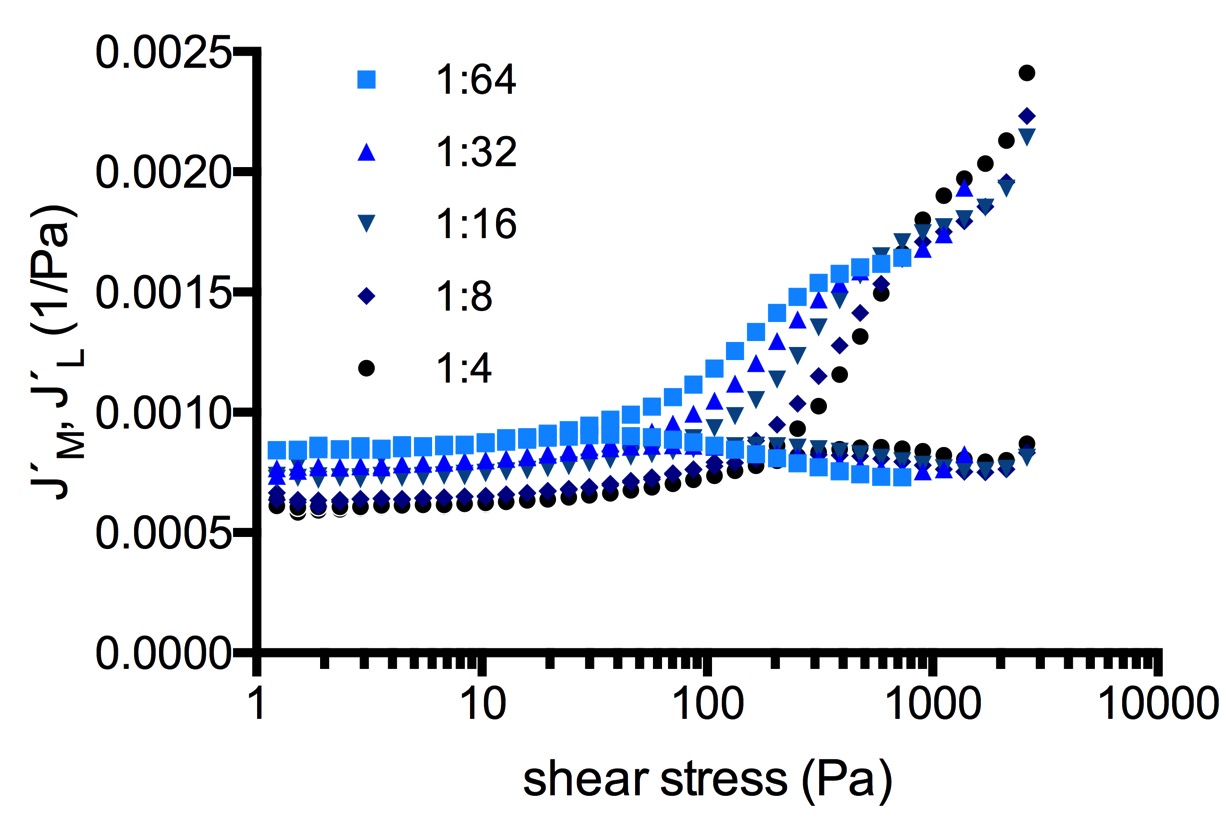

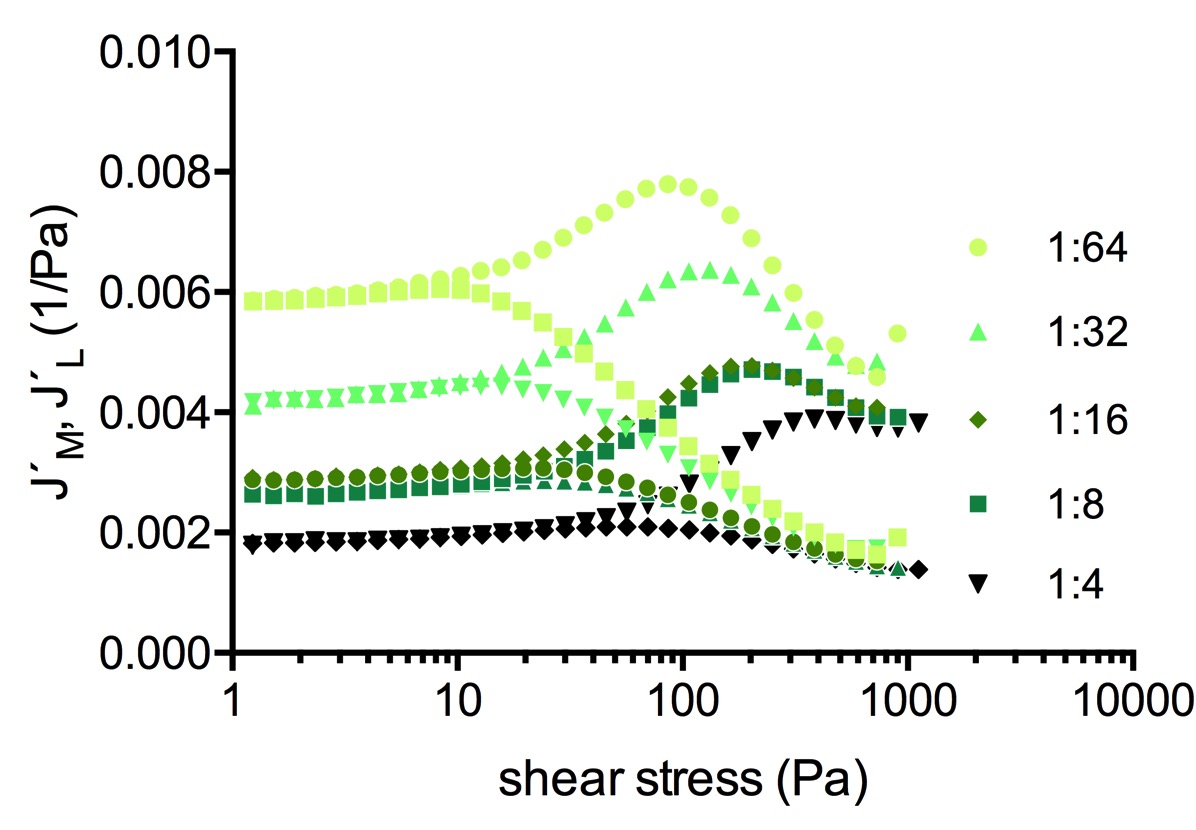

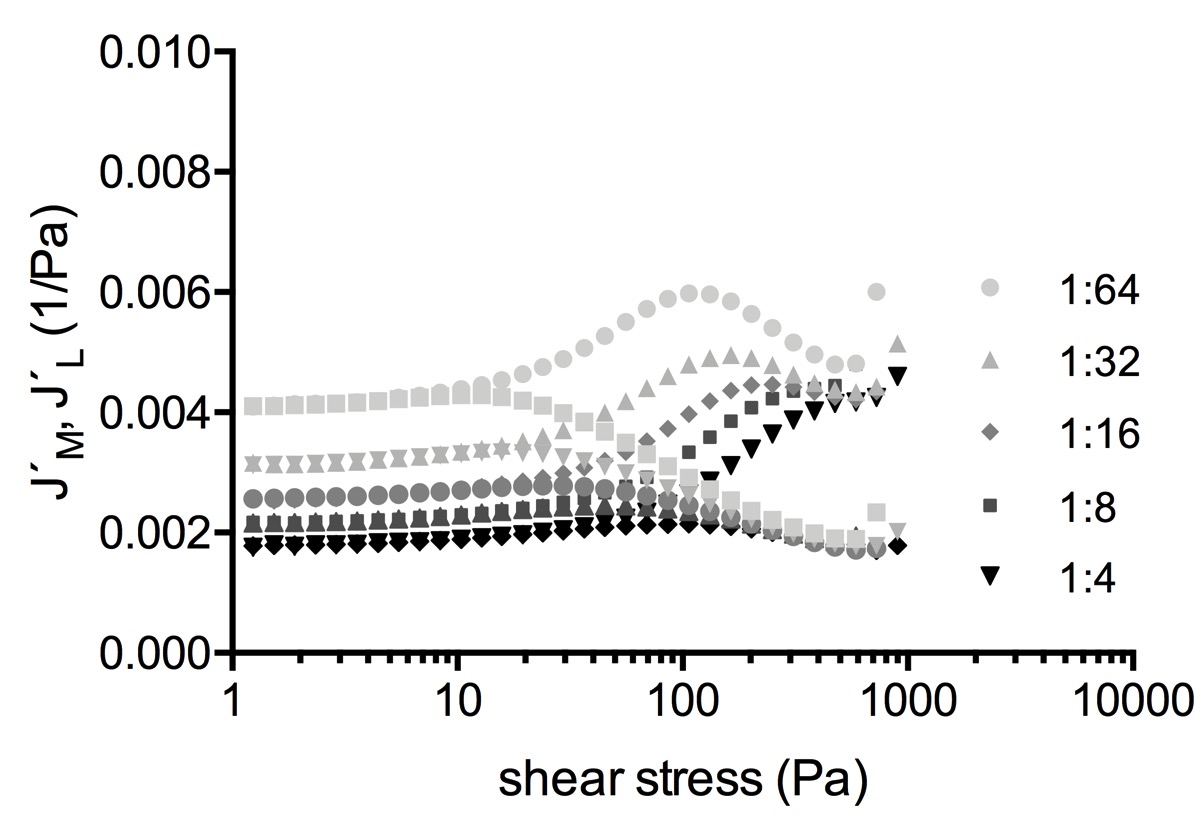


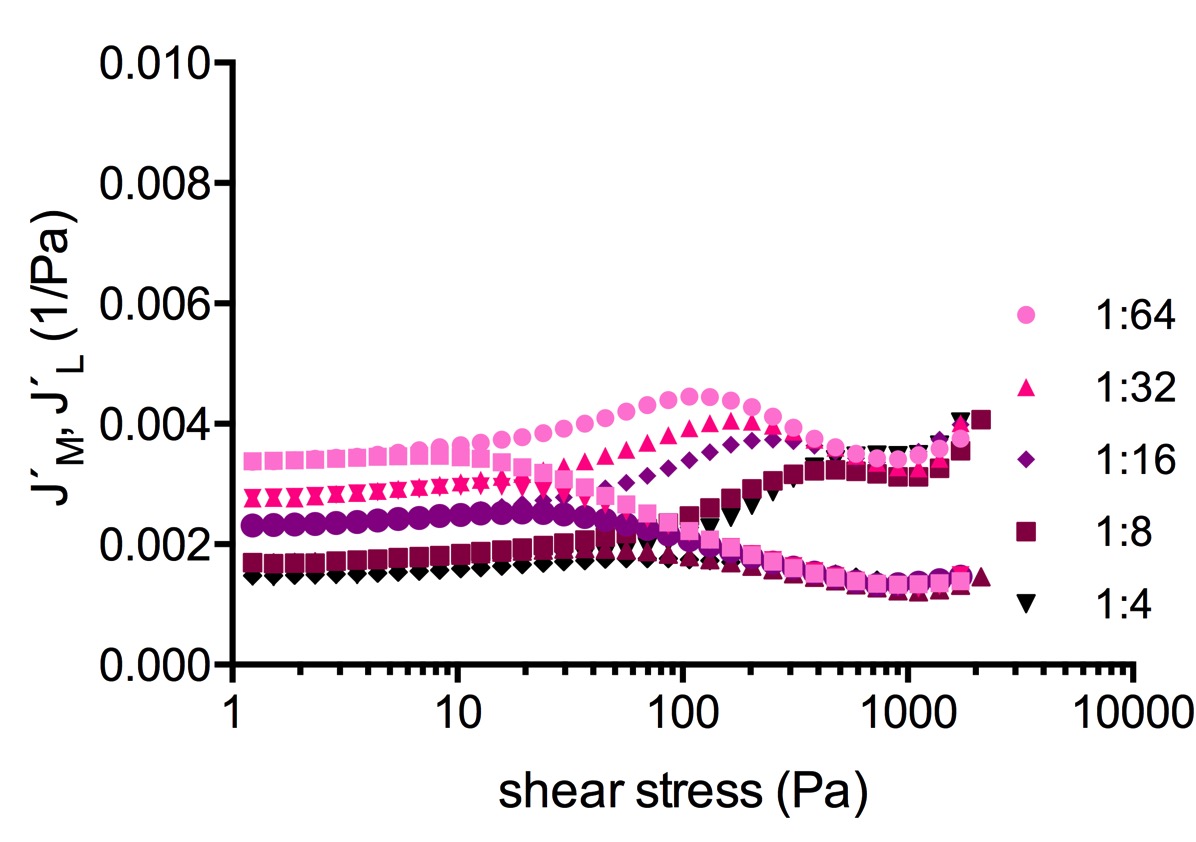


**Supportive Figure 2:** compliances decrease with the addition of platelets in each species (a: human, b: cow, c: swine, d: rat, e: horse). The compliance curves are species-specific. Stress thresholds shift with the platelet count in each species. Cow clots display only a tentative maximum for J’_M_.
