# Supplementary material for: Blood Clot Phenotyping by Rheometry: Platelets and Fibrinogen Chemistry Affect Stress-Softening and -Stiffening at Large Oscillation Amplitude
Source: Molecules. 2020 Aug 26;25(17):3890. doi: 10.3390/molecules25173890 (PMC7503632; doi:10.3390/molecules25173890)
Supplement: Supplementary file 1 [file molecules-25-03890-s001.zip › Supportive Figure 4.docx]

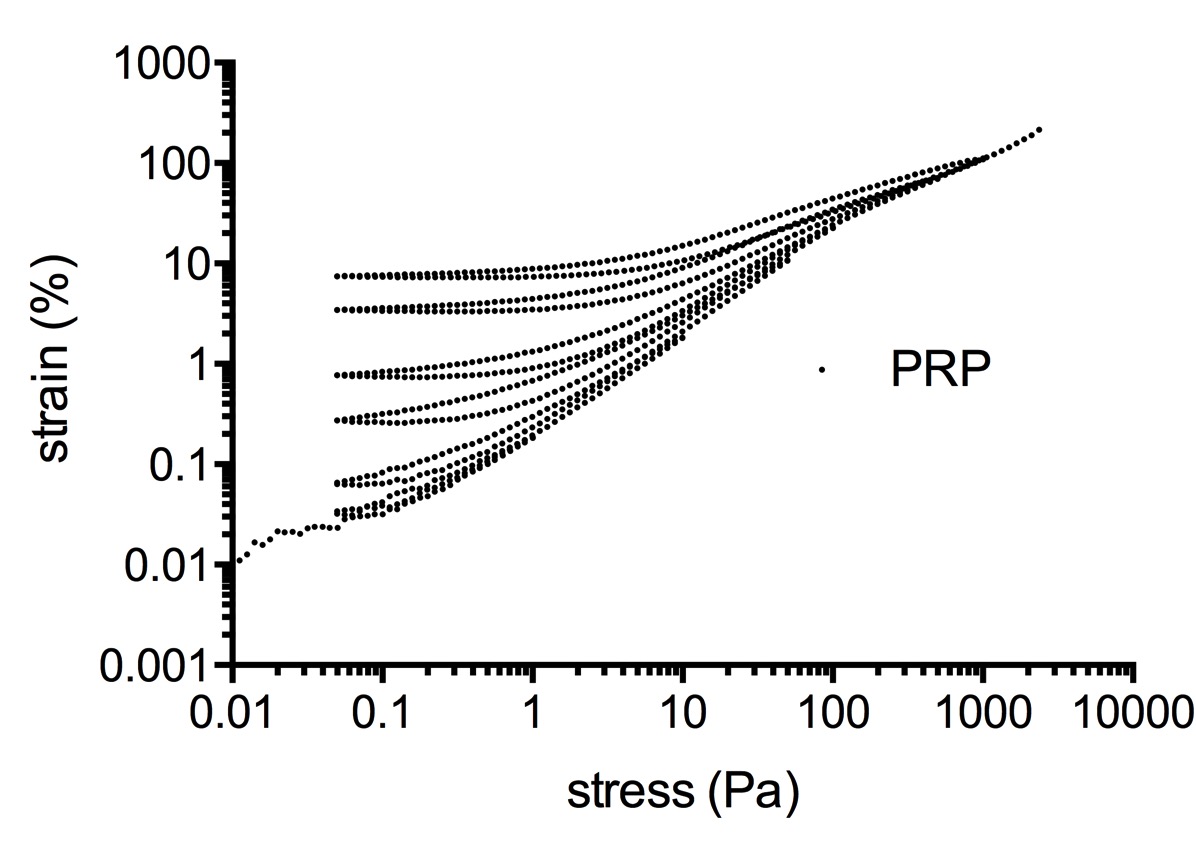

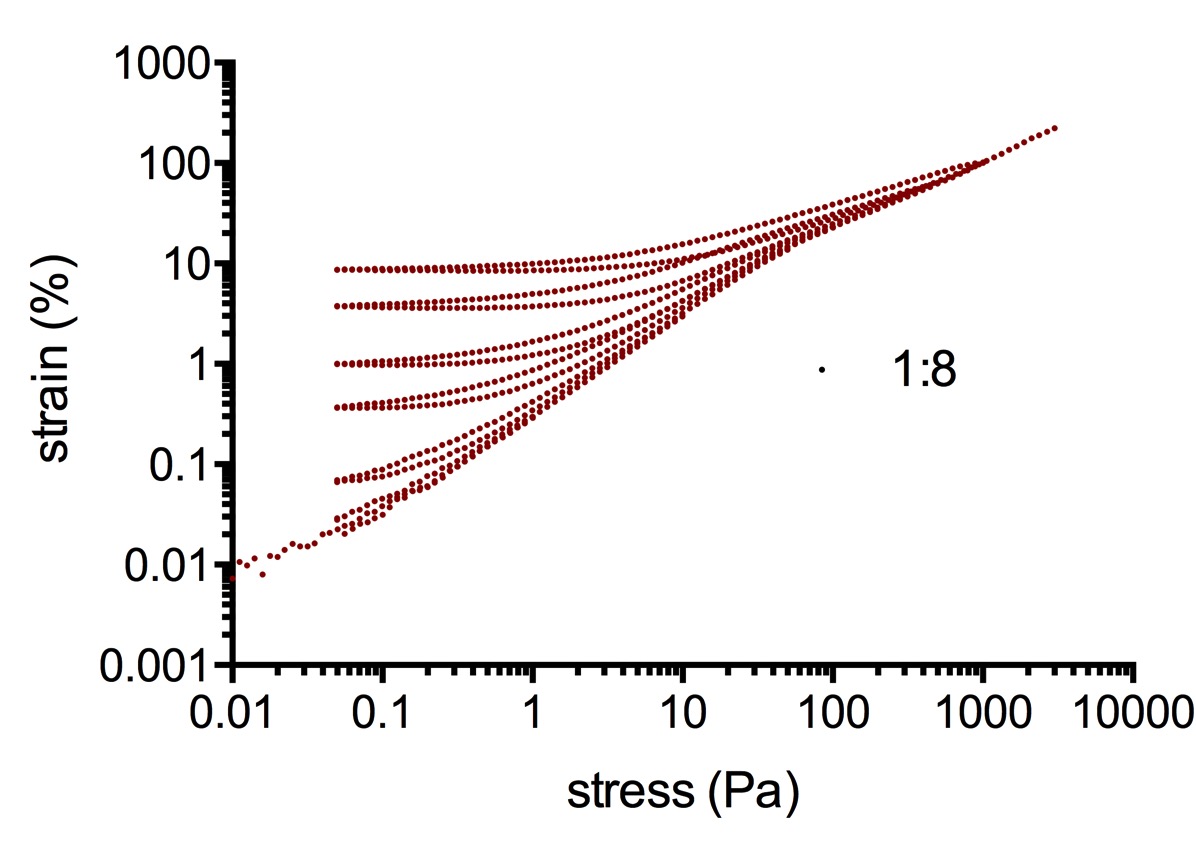

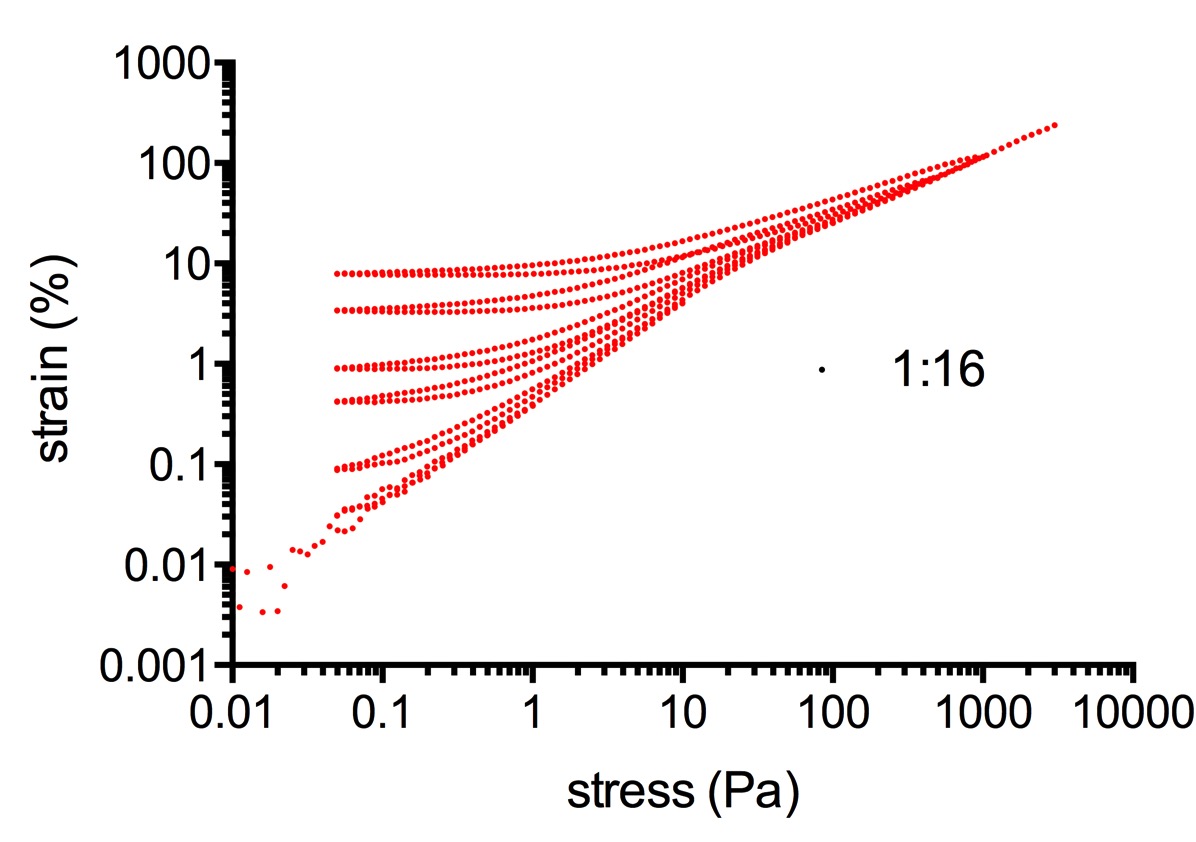

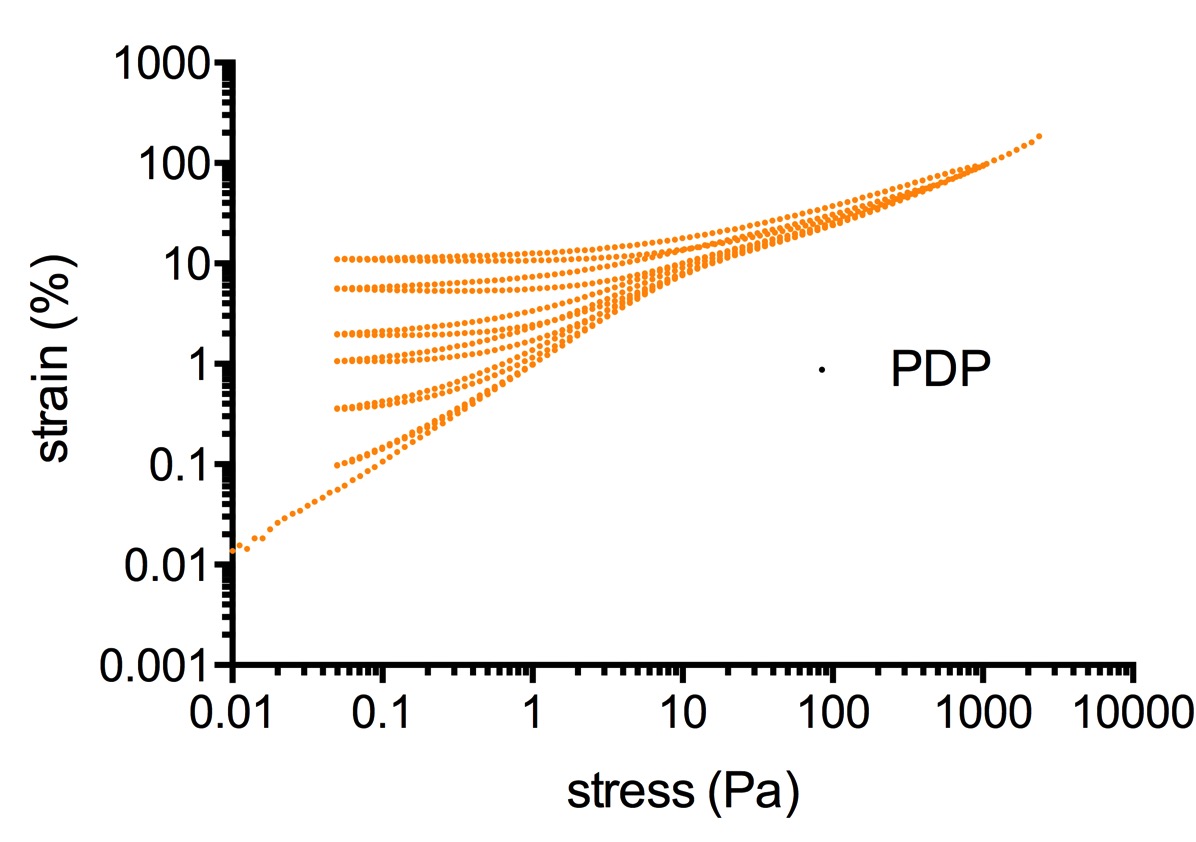


**Supportive Figure 4:** recurrent static stress loading and relaxation of human clots containing various platelet concentrations according to reference.


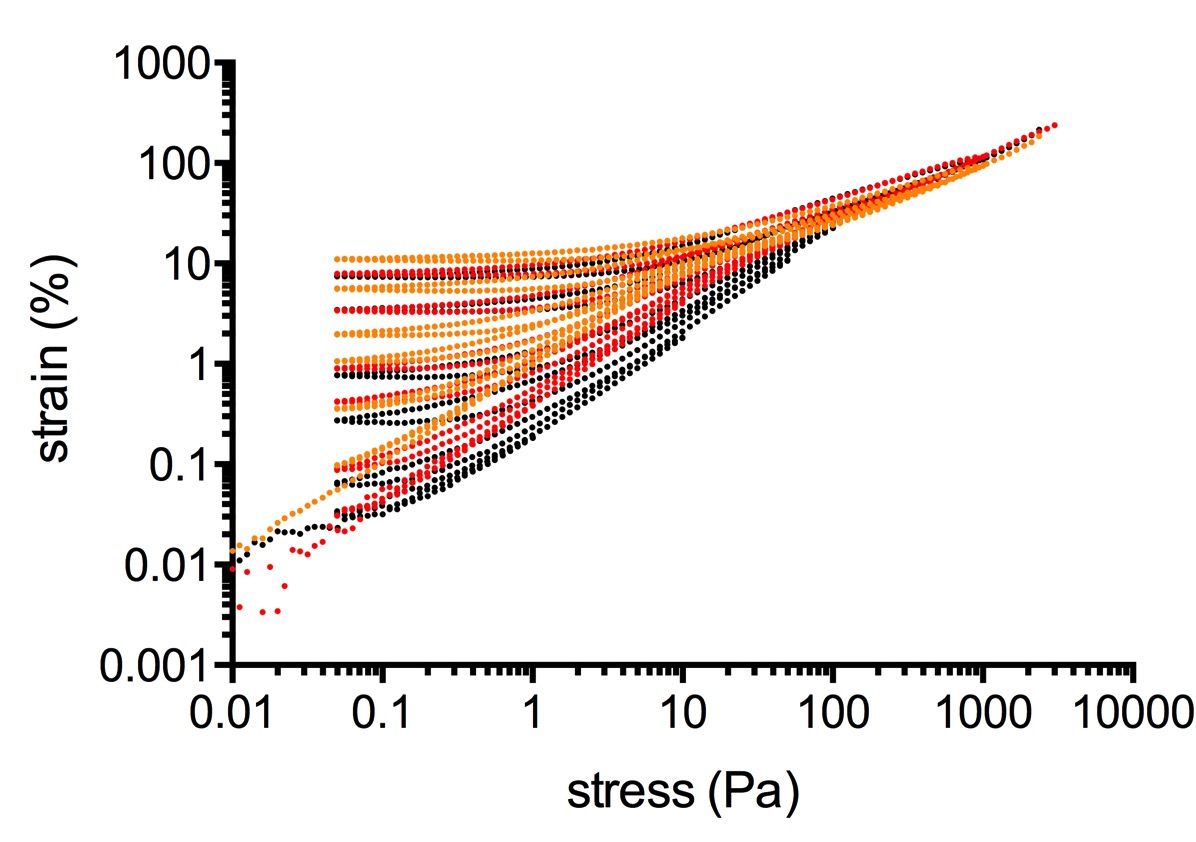


Reference: N. A. Kurniawan, B. E. Vos, A. Biebricher, G. J. L. Wuite, E. J. G. Peterman, G. H. Koenderink. Fibrin networks support recurring mechanical loads by adapting their structure across multiple scales. Biophys J 2016;111:1026–1034
